# Towards the realization of dynamically adaptable manufacturing automation systems

**DOI:** 10.1098/rsta.2020.0365

**Published:** 2021-10-04

**Authors:** Robert Harrison, Daniel Vera, Bilal Ahmad

**Affiliations:** Automation Systems Group, WMG, University of Warwick, Coventry CV4 7AL, UK

**Keywords:** automation, manufacturing, systems, optimization, dynamic, adaptable

## Abstract

The transition from traditional to truly smart dynamically adaptable manufacturing demands the adoption of a high degree of autonomy within automation systems, with resultant changes in the role of the human, in both the manufacturing and logistics functions within the factory. In the context of smart manufacturing, this paper describes research towards the realization of adaptable autonomous automation systems from both the control and information perspectives. Key facets of the approach taken at WMG are described in relation to human–machine interaction, autonomous approaches to assembly and intra-logistics, integration and dynamic system-wide optimization. The progression from simple distributed behavioural components towards autonomous functional entities is described. Effective systems integration and the importance of interoperability in the realization of more distributed and autonomous automation systems are discussed, so that operational information can propagate seamlessly, eliminating the traditional boundary between operational technology and information technology systems, and as an enabler for global knowledge collection, analysis and optimization.

This article is part of the theme issue ‘Towards symbiotic autonomous systems'.

## 1. Introduction

A number of trends are observable in automation from the complete automation paradigms of the 1980s and 1990s which were high performance dedicated single-use systems, to robotic systems in the 1990s and early 2000s, through modular flexible automation, and now towards autonomous decentralized self-organizing systems. The general trend is towards greater flexibility, and in the context of autonomous decentralized systems, we are in the paradigm of Industry 4.0 and digitalization where (at its heart) optimization becomes core to the realization of the smart factory.

A number of research groups are looking at the potential for self-organizing manufacturing systems [1]. Such approaches are regarded as vitally important to achieve more flexible and reconfigurable manufacturing [2,3]. However, such systems currently remain largely academic. Knowledge representation for flexible manufacturing is receiving a high degree of attention with the goal of enabling plant operations to become better optimized, and more knowledge intensive, distributed and collaborative in nature [4,5].

The transition from traditional to truly smart, dynamically adaptable, manufacturing demands the adoption of a high degree of autonomy within automation systems, with resultant changes in the role of the human, in both the manufacturing and logistics functions within the factory. Automation should be sympathetic with its operators and sustainable as well as profitable. In the context of smart manufacturing, this paper describes research towards the realization of adaptable, autonomous, automation systems from both the control and information perspectives.

Adaptive automation can only be effectively realized through a coherent system-of-systems approach where many distributed subsystems need to coexist, effectively communicate, and be mutually optimized in order for the much-vaunted potential of industrial digitalization to be attained.

As reported by Ng *et al*. [6–8], there is a general misconception that Industry 4.0 is all about digitalization, the Internet of Things, Big Data, smart machines, human–robot collaborations and all kinds of advanced manufacturing and their supporting IT technologies. Ng concludes that a careful reading of some definitions of Industry 4.0 [9] reveals that the ultimate goal of Industry 4.0 is actually to ‘optimize the entire value chain’, from gathering customer requirements to the design, manufacturing, deliveries, as well as after-sale services and recycling, in order to improve the overall competitiveness of manufacturing companies. Optimization technologies, therefore, play a significant role in making a modern industry competitive at various levels.

Engineering tools also have a major role to play in effectively supporting the lifecycle from design through deployment to operational optimization and to change management [10]. From a user perspective, such tools need to be pervasive, usable throughout the system lifecycle and capable of supporting the design, deployment and operational phases directly from a common engineering model, or digital twin as it has become known [11]. Such models are key enablers for lifecycle knowledge capture, configuration, optimization and reuse of modular manufacturing systems [12]. Many agent-based, service-oriented and component-based technologies, including IEC61499 distributed function block-based systems, are capable of providing suitable runtime infrastructures/platforms [13], but the supporting methodologies are often weak, fragmented and poorly aligned to user requirements [14]. A principal enhancement of IEC 61499 is that it enables a control system to be modelled and developed as a single integrated system, yet deployed as a distributed system [5].

Dynamic adaptability thus sees a distributed combination of autonomous actors in a self-organizing and adaptable system-of-systems with emergent behaviours. In the manufacturing context, this needs to be augmented by the overarching optimization of the overall production system. This paper considers the overall concepts in a symbiotic context via consideration of a set of complimentary research activities at the University of Warwick.

## 2. WMG research towards dynamically adaptable manufacturing automation

Dynamically adaptable manufacturing automation (DAMA) research at WMG aims to realize a consistent model-driven approach integrating and distributing both controls and analytics to yield more optimized production systems, which can be dynamically adapted and consistently engineered and changed with maximum robustness and security. Making KPIs visible to machine operators and managers achieving a consistent ‘single source of truth’ is a key objective of the work.

Having established the context for the work, the remainder of this paper will now look at four key facets of the approach taken at WMG in relation to (i) better human–machine interaction, (ii) autonomous approaches to assembly and intra-logistics within the production context, (iii) the need for the integration of these autonomous systems, and (iv) to support the dynamic optimum combination of local autonomous activities, combined with global optimization, to meet often complex performance objectives, e.g. with regard to operational efficiency, energy and sustainability goals in the context of the circular economy and the emerging drivers for industrial performance in a Net Zero world.

The Automation Systems Group at WMG is building a set of full-scale demonstrators to enable the realization of DAMA and its effective showcasing to the industry in the context of real production problems. These demonstrators are modular and reconfigurable systems and hence the applications can be progressively changed as new requirements emerge. The application scenarios being trialled all relate to real-use cases with industry partners.

**Figure 1.**
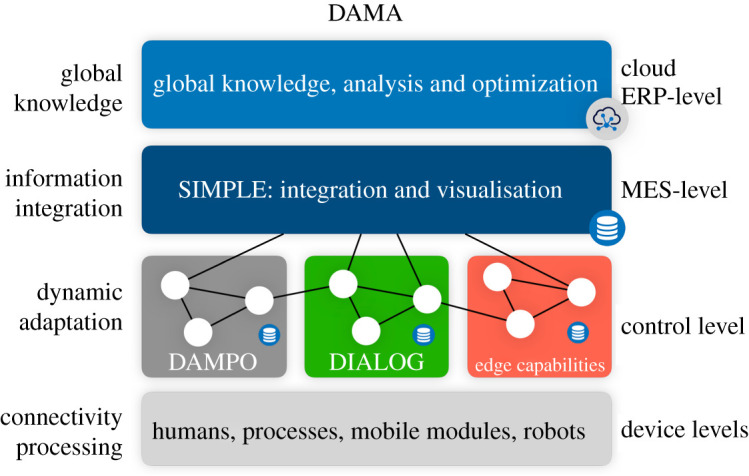
Relationship between DAMA-related research activities at WMG. (Online version in colour.)

Figure 1 illustrates the relationship between the featured research projects at WMG. The digitally assisted manufacturing process optimization (DAMPO) and dynamically integrating manufacturing automation with internal logistics (DIALOG) projects relate to autonomous systems for human–machine interaction, assembly and logistics. The approach is extendable through adding further control-oriented edge capabilities, e.g. distributed scheduling and decision support functions. The smart information platform and ecosystem for manufacturing (SIMPLE) project supports integration and visualization, and above this sits global knowledge analysis and optimization. The projects and perspectives are described in more detail in the following sections.

## 3. Human–machine interaction and interfacing

### (a) Introduction

As highlighted by Sundblad, the debate within manufacturing about whether technology will completely replace people is interesting, but it is the wrong debate [15]. The discussion should be about the concept of human–machine symbiosis, the mutually beneficial relationship between humans and technology, and how machines and software can intelligently and physically increase the productivity of the systems to be more than that of human or machine alone. The goal is to optimize and get the best out of both humans and robots in a combined ecosystem working symbiotically. For example, humans can easily deal with the unexpected, take complex decisions quickly, sense and process a large variety of data and have great dexterity for complex handling tasks. Robots, and automation more generally, can deal with high precision repetitive tasks without getting tired or bored and can quickly and reliably sense and process a large number of inputs and data.

### (b) Digitally assisted manufacturing process optimization

Autonomous systems that require human collaboration (or intervention) are known as symbiotic autonomous systems. However, introducing humans in the loop presents technical challenges. The DAMPO project focuses on developing an innovative solution (combination of hardware and software) and engineering methods to enhance the design and monitoring of complex and safety critical manual assembly processes in the manufacturing industry. The DAMPO project development relies on the effective integration of optical position tracking and data capture technology, methods and solutions for mass data visualization and analytics, and engineering methods for manual operations design optimization.

The vision of DAMPO is to achieve seamless interaction between the digital systems and human operators in order to effectively augment manual operations. The key objectives of the project are to (i) enable unobtrusive and effective data capture of processes carried out by human operators, (ii) provide contextualized, structured and, when needed, near real-time information feedback to the operator and (iii) support continuous data-driven process optimization in view of improving both process effectiveness (reduced time, improved quality and consistency) and operator well-being (ergonomics, cumulated fatigue).

The data capture systems deployed and tested include active and passive marker-based optical pose tracking systems, marker-less 2D/2.5D/Lidar camera systems and IMU cluster systems. In order to minimize process interferences and deployment constraints, markers and IMUs when used, are fitted on various components in the station/cell (e.g. tooling, fixtures, pallets and product) rather than on the operators. These systems allow capture of both operators' related data (e.g. skeleton pose and joints position tracking) and process-related data (e.g. fixtures/tools/parts location detection and tracking). Inside-out relative location tracking using tool-mounted camera and image processing is also being investigated and developed.

**Figure 2.**
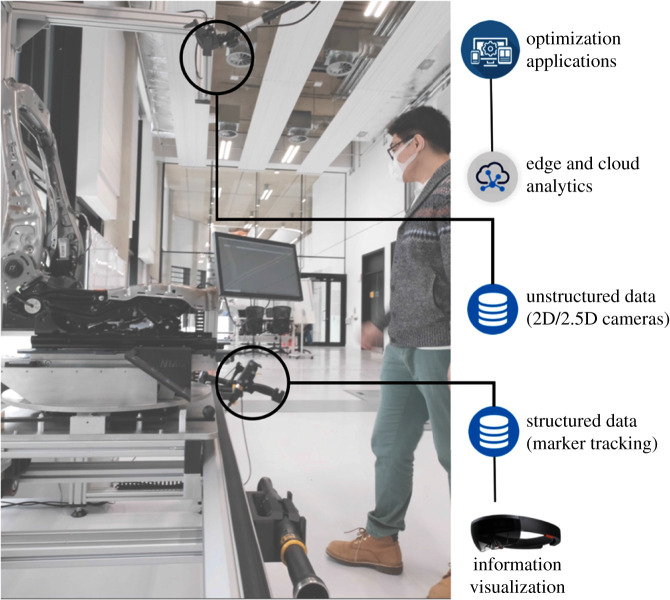
DAMPO manual operation tracking testbed. (Online version in colour.)

The DAMPO project adopts a holistic and data-driven approach to the optimization of manual operations. Data processing and analytics functions are developed to support ergonomic and cumulative fatigue-oriented optimization of operator work, station and process layouts optimization, as well as supporting quality assurance and process traceability. The functions rely on the processing and evaluation of instant data streams (e.g. instant ergonomic evaluation of current posture), as well as on the analysis of historical datasets. DAMPO focuses on developing unsupervised machine-learning optimization models using a combination of specific datasets (e.g. specific period of time, process and product types) in order to support station layout, processes, manual operations and potentially also product designs. The project testbed is shown in figure 2.

The DAMPO project includes the development of information delivery and presentation methods tailored to specific users and to the interactions required with the system. Screen-based HMIs and headset-based AR application are used to deliver contextual information back to the operators (e.g. process steps validation feedback, guidance and safety-related information). To further develop the interaction between digital system and human operator, AR is used in the DAMPO project to develop a solution allowing the operator to identify specific product features and attach contextual information. This system was primarily developed to enable in-process and in-line labelling of defects (defect localization and type labelling), which effectively allows quality technicians to build the dataset required to train a deep learning-based defect detection model. This highlights the potential of human/digital system symbiosis to define and develop new roles for operators on the shop floor and to reduce the overhead of workflows associated with the deployment and use of data-driven methods; offline labelling of two-dimensional images required for the training of supervised machine-learning model in this case.

## 4. Autonomous systems for assembly and intra-logistics

### (a) Introduction

Many emerging manufacturing processes now require the flexible integration of people working cooperatively with robots and autonomous guided vehicles, e.g. to closely combine assembly automation with materials handling. The aim is to minimize non-value adding activities within adaptable processes, so that the product variety and volumes can be dynamically changed.

With the recent shift towards more and more online trading, there has been an explosion in the need for logistics, and the biggest challenge facing the logistics industry today is labour availability. The mass arrival of robotics in logistics is no longer in question. It is happening now and the widespread presence of robots in warehouses is becoming a reality, and in turn there will be widespread utilization of robots in the intra-logistics context of manufacturing automation, with the blurring of the boundaries between internal logistics systems and manufacturing automation systems in flexible and novel configurations working alongside human operators.

From relatively simple pick-and-place operations, there is a progression now to more intelligent capabilities. For example, to handle a range of tasks where different tools are required, aided by the evolution of modularity in the robotic systems and the configuration of different transport systems to allow such autonomous systems to be used for a wide variety of tasks, in a variety of environments.

### (b) Dynamically integrating manufacturing automation with internal logistics

The DIALOG research project at WMG addresses the fact that much of manufacturing and indeed industry more widely operates in a far from optimal manner in the face of volatile customer preferences and often unforeseen disturbances.

The vision is for practical, dynamically adaptable automation systems that are readily scalable. Further to this, the concept is to enable a new form of low-cost manually operated intra-logistics systems that can be readily and progressively automated through the addition of autonomous mobile robots (AMRs).

The application focuses on integrating this system of systems within a generic infrastructure and related configuration tools, with use cases in the automotive seat assembly, riveted body assembly fastening, battery module and pack production, horticulture, and food and drink sectors. These will be oriented around the application of a generic approach based on the use of a new modular AMR system, and related integration and engineering tools for their deployment, integration and optimization. There are many innovative aspects to this work in the context of the role of machine operators and the human-centric perspectives on such systems of systems. There are opportunities to scale such systems through the flexible interplay of autonomous production movements combined sympathetically with manual operations with the operators guided by suitable tracking and traceability technologies.

The DIALOG project provides a modular platform and a generic framework for the deployment and optimization of modular plug-and-play AMR-based intra-logistic system on production shop floors to realize dynamic adaptability from both physical and control perspectives. The project exploits the use of modular trolleys with standard mechanical and electrical interfaces to enable towing of a wide range of trolleys with AMRs for various applications (e.g. pallet transportation). To communicate and integrate with automation systems, control functions are encapsulated and hence can be deployed and (re)configured as needed without the need to reprogram the entire system controls and communication functions. The combination of this hardware and software Lego-like plug-and-play capability allows for re-design and re-configuration of the logistics system as new requirements emerge.

**Figure 3.**
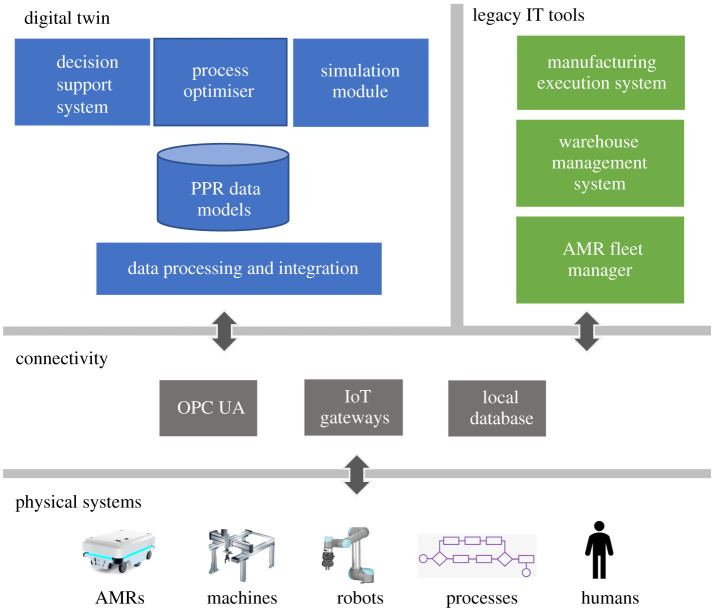
DIALOG integration framework for enabling dynamic optimization of shop floor intra-logistics. (Online version in colour.)

The integration framework for the DIALOG project is shown in figure 3. This integration framework is based on a data-driven approach which makes use of real-time product–process–resource (PPR) data to feed into the digital twin of the system and integrates with proprietary fleet managers, manufacturing execution systems and warehouse management systems. The framework enables dynamic (re)optimization of the intra-logistics in real-time through closed-loop integration with a digital twin of the intra-logistics.

In the digital twin space, optimized scheduling is realized through the use of what-if engine and discrete event simulation (DES) of the physical resources coupled with optimization algorithms. The decision support module is a real-time information delivery gateway and provides an interface for operators to interact with the system to predict the performance of the overall system. The decision support module is coupled with the simulation and optimizer modules and uses an inference engine to help operators in making informed decisions. Further details can be found in [16,17].

## 5. Integration of distributed control, information and events

### (a) Introduction

Previous work by the automation systems group at WMG on the knowledge-driven configurable manufacturing and other related projects has pioneered a component-based approach to reconfigurable production systems [10]. This resulted in a system architecture and associated engineering tools capable of building and deploying such systems. These tools support the paradigm of cyber-physical systems engineering with physical and digital representations of the system components or assets created and maintained throughout their lifecycle. A growing body of complementary international work in this domain, which is now commonly referred to as Industry 4.0, has seen the emergence of reference architectures and standards for such systems. Most notable in this context are the RAMI 4.0 and Industrial Internet Consortium reference architectures [18].

The use of a component-based approach for automation system development and implementation draws inspiration from component-based development (CBD) concepts and uses a distributed control methodology to encapsulate standard control functionality into common automation elements known as components. The manufacturing automation system can then be developed around these pre-build components by configuring them according to the desired application behaviour [13]. The CBD approach has been regarded as the answer for organizations to manage complexities and adapt to changes rapidly [19]. Instead of building a system from scratch, the CBD approach facilitates system development using previously developed system elements. It encourages and enables system development by building on and reusing past experiences and knowledge [20].

Communication between distributed components can be realized in a variety of ways, e.g. using either proprietary or open networking systems and protocols. For example, the FP6 SOCRADES project involving WMG evaluated several service-oriented architecture solutions, applicable at the device level integration, including DPWS and OPC-UA, in the context of manufacturing automation. To address the integration of very large numbers of subsystems and devices, the subsequent AESOP project took its roots in previous work in several European collaborative projects (ITEA SIRENA; FP6 IP SOCRADES; VINNOVA-Sweden, 2007–2009), which demonstrated that embedding web services at the device level and integrating these devices with systems at upper levels of an enterprise architecture was feasible.

### (b) Smart information platform and ecosystem for manufacturing

While the previously described projects notably advanced connectivity both with, and between, autonomous devices, the ability of end-users to simply, consistently and reliably integrate key information across diverse production systems is currently a major barrier to productivity gains in industry and is thus the focus of the current SIMPLE research project at WMG.

A fundamental challenge in deploying effective digital capabilities is the integration of systems within and across the operational technology (OT) and information technology (IT) layers of manufacturing organizations. This integration is typically currently hindered by either a lack of identifiable frameworks, design methods and engineering tools (technology diversity and fragmentation) or by the cost, complexity and long-term strategic lock-in associated with the adoption of proprietary and monolithic commercial solutions. The SIMPLE project applies innovative software technology to address this need, proving the resultant solution in a practical and widely applicable industrial form.

**Figure 4.**
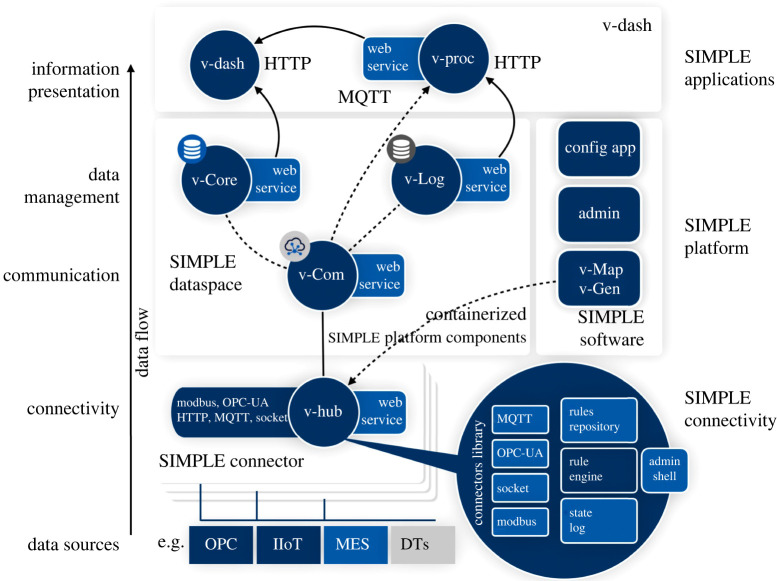
SIMPLE platform architecture and core components. (Online version in colour.)

Data pipelines and information flows are essential to the architectural and operational integration of various systems within and between manufacturing organizations. The SIMPLE platform development, figure 4, aims at rationalizing the collection of manufacturing data across manufacturing sectors: The project focuses specifically on the design of robust and scalable data transport, data management and data processing architectures, and on the integration of shop floor level (OT), inter (i.e. IT-level) and extra-organization (e.g. cloud-level) data systems. A key aspect of the project and the core element of the SIMPLE platform is the definition of production data models for discrete manufacturing. The SIMPLE platform implements a PPR data model that provides manufacturing organizations with a template for the complete data pipeline, i.e. the collection, management, processing and visualization of manufacturing and production data.

The effectiveness of data-driven monitoring and optimization systems directly depends on the quality of the datasets. The SIMPLE PPR model promotes systems and methods that focus on collecting complete sets (product process and resource data types) of high-quality data rather than large sets of heterogeneous, unstructured and uncontextualized data (‘Big Data/Data Lake’ approach). The SIMPLE platform PPR data models support the definition of data metrics and quality assessment functions that contribute to inform (and educate) production and manufacturing engineers on the quality of the data collected from the processes and systems in the shop floor.

The SIMPLE platform specification guides the implementation of the PPR model across various types of databases and data management systems. Currently, relational (v-Core), NoSQL and time series databases (v-Log) configurations are being developed for various industrial use cases, and the future project phase will focus on extending the SIMPLE data model to include knowledge representation (taxonomies, semantic representation) and their implementation in graph databases.

The SIMPLE platform implementation also supports the integration of industrial and IT level networks and communication protocols. Specific platform components (v-Hub connectivity toolbox and v-Com pub/sub broker) provide IT/OT and IT/Cloud connectors to support the deployment of robust and scalable data workflows.

The application layer of the platform SIMPLE provides a set of pre-defined (i.e. aligned to the PPR data model) data processing and analytics functions, as well as the corresponding production monitoring dashboards, to support common manufacturing requirements and use cases: e.g. monitoring and predictive analysis of production-related data such as process performances, quality, product traceability and maintenance-related information. Various software modules support the deployment configuration, maintenance and general administration of the platform's components.

## 6. Optimization

Many research projects and industrial companies are working towards the realization of Industry 4.0 and the self-organizing factory. In this context, several leading academics have recently commented on the fact that the real core concept of Industry 4.0 is related to optimization, as discussed in §1. Once the necessary connectivity has been realized to enable the collection of good quality data capable of forming the basis of actionable insights derived from the multitude of devices, the challenge is dynamically optimizing production through both local and global adaptations.

Decision support and optimization of manufacturing processes in real-time are demanding tasks. Simulation-based offline optimization techniques are increasingly used in industry today to perform what-if analysis based on foresight of possible disruptions/changes on shop floors. However, such an approach only partially improves system performance. There is a need for a real-time dynamic optimization approach that couples simulation models with machine-learning and optimization algorithms.

To address this problem, WMG is carrying out research in collaboration with Polytechnic Institute of Braganca using a digital twin-based optimization approach coupled with AI and decision support systems. This research is using a PPR data model developed in the SIMPLE project [21] to feed relevant real-time data to the simulation module.

**Figure 5.**
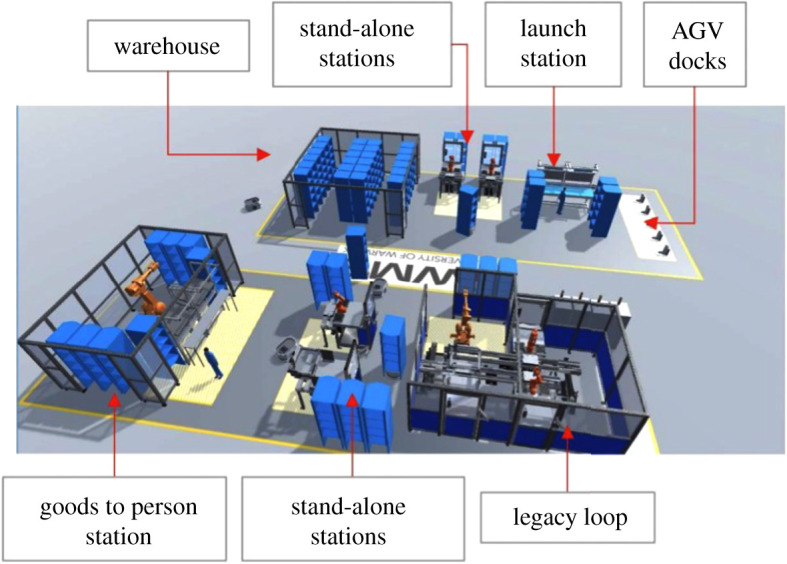
Layout of the IML demonstrator at WMG. (Online version in colour.)

A proof-of-concept demonstrator has been developed at WMG using the integrated manufacturing and logistic (IML) rig at WMG which is a prototype automotive battery module and pack assembly line; figure 5.

Optimization is carried out based on target production and real-time data collected from IML for two different aspects: (a) AMR dispatch scheduling optimization to dynamically optimize line-side supply and pallet transportation and (b) AMR energy management to optimally charge the AMR batteries with minimum impact on production schedules. Both of the optimization problems are complex in nature as the scheduling decision is based on multiple variables and multiple criteria.

The developed framework for optimization is based on three main components: a simulation module, an analytics module and a decision support module. The simulation module is essentially a DES of the facility developed in Witness which is coupled with a what-if simulation engine to simulate various scenarios. The analytics module is sub-divided into optimization and machine-learning modules. The optimization module uses mixed-integer nonlinear programming using a genetic algorithm integrated with the DES model. The ML module learns from the previous decisions taken and their resultant impact on KPIs of the system. The decision support module provides an interface for users to interact with the analytics and simulation modules to perform what-if analysis to determine optimal system variables for (re)scheduling of AMRs. The interface allows users to rate decisions which can be used to determine the trust level of decisions.

Beyond simulation-based and AI-based process optimization, and as highlighted by Ng, digitization methods and tools supporting the design and improvement of next-generation manufacturing systems must be developed to take into account all levels of operation and their complex interactions, in order to provide strategic and tactical support, and operational decision-making in a multi-criteria context is needed, as exemplified by their knowledge-driven optimization research programme [6,22].

## 7. Conclusion

A set of inter-related research activities at WMG have been used to describe progress towards the realization of DAMA systems. There is a significant opportunity to create a unified approach to autonomous control and analytics, both locally and globally. The DAMA vision is to enable adaptable manufacturing systems to be engineered efficiently, incorporating analytics and supporting both local and global optimization. It envisages a new paradigm of integrated data models spanning the complete lifecycle to effectively support dynamic adaptability, establishing effective lifecycle digital twins, where such models are directly deployable to the shop floor and maintained throughout the production system and associated product lifecycles.

Particular challenges now relate to achieving globally optimized performance while at the same time allowing dynamic adaptation to occur within distributed autonomous systems, enabling self-optimization while at the same time adapting to global system challenges. For example, in relation to energy efficiency and sustainability, and in the context of larger ecosystems, extending across supply chains and throughout complete lifecycles in a circular economy.

## Data accessibility

This article has no additional data.
